# Matching Aerial Images to 3D Building Models Using Context-Based Geometric Hashing

**DOI:** 10.3390/s16060932

**Published:** 2016-06-22

**Authors:** Jaewook Jung, Gunho Sohn, Kiin Bang, Andreas Wichmann, Costas Armenakis, Martin Kada

**Affiliations:** 1 Department of Earth and Space Science and Engineering, York University, 4700 Keele Street, Toronto, ON M3J 1P3, Canada; gsohn@yorku.ca (G.S.); kiinbang@yorku.ca (K.B.); armenc@yorku.ca (C.A.); 2 Institute of Geodesy and Geoinformation Science (IGG), Technische Universität Berlin, Straße des 17. Juni 135, 10623 Berlin, Germany; andreas.wichmann@tu-berlin.de (A.W.); martin.kada@tu-berlin.de (M.K.)

**Keywords:** registration, 3D building models, aerial imagery, geometric hashing, model to image matching

## Abstract

A city is a dynamic entity, which environment is continuously changing over time. Accordingly, its virtual city models also need to be regularly updated to support accurate model-based decisions for various applications, including urban planning, emergency response and autonomous navigation. A concept of continuous city modeling is to progressively reconstruct city models by accommodating their changes recognized in spatio-temporal domain, while preserving unchanged structures. A first critical step for continuous city modeling is to coherently register remotely sensed data taken at different epochs with existing building models. This paper presents a new model-to-image registration method using a context-based geometric hashing (CGH) method to align a single image with existing 3D building models. This model-to-image registration process consists of three steps: (1) feature extraction; (2) similarity measure; and matching, and (3) estimating exterior orientation parameters (EOPs) of a single image. For feature extraction, we propose two types of matching cues: edged corner features representing the saliency of building corner points with associated edges, and contextual relations among the edged corner features within an individual roof. A set of matched corners are found with given proximity measure through geometric hashing, and optimal matches are then finally determined by maximizing the matching cost encoding contextual similarity between matching candidates. Final matched corners are used for adjusting EOPs of the single airborne image by the least square method based on collinearity equations. The result shows that acceptable accuracy of EOPs of a single image can be achievable using the proposed registration approach as an alternative to a labor-intensive manual registration process.

## 1. Introduction

In recent years, a number of mega-cities such as New York and Toronto have built-up detailed 3D city models to support the decision-making process for smart city applications. These 3D models are usually static snapshots of the environment and represent the status quo at the time of their data acquisition. However, cities are dynamic systems that continuously change over time. Accordingly, their virtual representations need to be regularly updated in a timely manner in order to allow for accurate analysis and simulation results that decisions are based upon. In this context, a framework for continuous city modeling by integrating multiple data sources was proposed by [1].

A fundamental step to facilitate this task is to coherently register remotely sensed data taken at different epochs with existing 3D building models. Great research efforts have already been undertaken to address the related problem of image registration. [2,3], e.g., give comprehensive literature reviews of relevant methods. Fonseca *et al.* [4] conducted a comparative study of different registration techniques for multisensory remotely sensed imagery. Although most of the existing registration methods have shown promising success in controlled environments, registration is still a challenging task due to the diverse properties of remote sensing data related to resolution, spectral bands, accuracy, signal-to-noise ratio, scene complexity, occlusions, *etc.* [3]. These variables have a major influence on the effectiveness of the registration process, and lead to severe difficulties when attempting to generalize it. Still, though a universal method applicable to all registration tasks seems impossible, the majority of existing method consists of the following three steps [2,5]:

- *Feature extraction*: Salient features such as closed-boundary regions, edges, contour lines, intersection points, corners, *etc.* are detected in two datasets, and used in the registration process. Special care has to be taken to ensure that these features are distinctive, well distributed and can be reliably observed in both datasets.
- *Similarity measure and matching*: The correspondences between features that are extracted from two different datasets are then found by a matching process. A similarity measure that is based on the attributes of the features quantifies its correctness. To be effective, the measure should consider the specific feature characteristics in order to avoid possible ambiguities, and to be accurately evaluated.
- *Transformation*: Based on the established correspondences, a transformation function is constructed that transforms one dataset to the other. The function depends on the assumed geometric discrepancies between both datasets, the mechanism of data acquisition, and required accuracy of the registration.

A successful registration strategy must consider the characteristics of the data sources, its later applications, and the required accuracy during the design and combination of the individual steps. Recent advancements of aerial image acquisition make direct geo-referencing for certain types of applications (coarse localization, and visualization) possible. If an engineering-level accuracy is needed, however, including continuous 3D city modeling, the exterior orientation parameters (EOPs) obtained through these techniques may need to be further adjusted. In indirect geo-referencing of aerial images, accurate EOPs are generally determined by bundle adjustment with ground control points. However, obtaining or surveying such points over a large-scale area is labor intensive, and time-consuming. An alternative method is to use other known points instead.

Nowadays, large-scale 3D city models have been generated for many major cities in the world, and are, e.g., available within the Google Earth platform. Thus, the corner points of 3D building models can be used for registration purposes. However, the quality of the existing models is often unknown and varies furthermore from building to building, which is the result from different reconstruction methods and data sources being applied. For example, LiDAR points are mostly measured within the roof faces and seldom at their edges, which often results in their boundaries and corner points to be geometrically inexact. Thus, the sole use of corner points from existing building data bases as local features can lead to matching ambiguities and therefore to errors in the registration.

To address this issue for the registration of single images with existing 3D building models, we propose to use two types of matching cues: (1) edged corner features that represent the saliency of building corner points with associated edges; and (2) context features that represent the relations between the edged corner features within an individual roof. Our matching method is based on the Geometric Hashing method, which is a well-known indexing-based object recognition technique [6], and it is combined with a scoring function that reinforces the context force. We have tested our approach on large urban areas with over 1000 building models in total.

### Related Work

Registration is an essential process when multisensory datasets are used for various applications such as object recognition, environmental monitoring, change detection, and data fusion. In computer vision, remote sensing and photogrammetry, this includes registrations between same source taken from different viewpoints at different times (e.g., image to image), between datasets collected with different sensors (e.g., image and LiDAR), and between an existing model and remotely sensed raw data (e.g., map and image). Numerous registration methods have been proposed to solve the registration problems for given environments, and for different purposes [2,3,4,7]. Regardless of data types and applications, the registration process can be recognized as a feature extraction, and correspondence problem (or matching problem) between datasets. Brown [2] categorized the existing matching methods into area-based, and feature-based methods according to their nature. Area-based matching methods use image intensity values extracted from image patches. They deal with images without attempting to detect salient objects. Correspondences between two image patches are determined with a moving kernel sliding across a specific size of image search window or across the entire other image by correlation-like methods [8], Fourier methods [9], mutual information methods [10], and others. In contrast, feature-based methods use salient objects such as points, lines, and polygons to establish relations between two different datasets. In feature matching processes, correspondences are determined by considering the attributions of the used features. In model-to-image registration, most of the existing registration methods adopt a feature-based method because many 3D building models have no texture information.

In terms of features, point features such as line intersections, corners and centroids of regions can be easily extracted from both models and images. Thus, Wunsch *et al.* [11] applied the Iterative Closest Point (ICP) algorithm to register 3D CAD-models with images. The ICP algorithm iteratively revises the transformation with two sub-procedures. First, all closest point pair correspondences are computed. Then, the current registration is updated using the least square minimization of the displacement of matched point pair correspondences. In a similar way, Avbelj *et al.* [12] used point features to align 3D wire-frame building models with infrared video sequences using a subsequent closeness-based matching algorithm. Lamdan *et al.* [6] used a geometric hashing method to recognize 3D objects in occluded scenes from 2D grey scale images. However, Frueh *et al.* [13] pointed out that point features extracted from images cause false correspondences due to a large number of outliers.

As building models or man-made objects are mainly described by linear structures, many researchers have used lines or line segments instead of points as features. Hsu *et al.* [14] used line features to estimate the 3D pose of a video where the coarse pose was refined by aligning projected 3D models of line segments to oriented image gradient energy pyramids. Frueh *et al.* [13] proposed a model to image registration for texture mapping of 3D models with oblique aerial images. Correspondences between line segments are computed by a rating function, which consists of slope and proximity. Because an exhaustive search to find optimal pose parameters was conducted, the method is affected by the sampling size of the parameter space, and it is computationally expensive. Eugster *et al.* [15] also used line features for real-time geo-registration of video streams from unmanned aircraft systems (UAS). They applied relational matching, which does not only consider the agreement between an image feature and a model feature, but also takes the relations between features into account. Avbelj *et al.* [16] matched boundary lines of building models derived from DSM and hyper-spectral images using an accumulator. Iwaszczuk *et al.* [17] compared RANSAC and the accumulator approach to find correspondences between line segments. Their results showed that the accumulator approach achieves better results. Yang *et al.* [18] proposed a method to register UAV-borne sequent images and LiDAR data. They compared building outlines derived from LiDAR data with tensor gradient magnitudes and orientation in image to estimate key frame-image EOPs. Persad *et al.* [19] matched linear features between Pan-Tilt-Zoom (PTZ) video images with 3D wireframe models based on hypothesis-verification optimization framework. However, Tian *et al.* [20] pointed out several reasons that make the use of line or edge segments for registration a difficult problem. First, edges or lines are extracted incompletely, and inaccurately so that ideal edges might be broken into two or more small segments that are not connected to each other. Secondly, there is no strong disambiguating geometric constraint, whereas building models are reconstructed with certain regularities such as orthogonality, and parallelism.

Utilizing prior knowledge of building structures can reduce the matching ambiguities, and the search space. 3D object recognition method from single image based on the notion of perceptual grouping, which groups image lines based on proximity, parallelism and collinearity relations, was proposed in [21]. Also, hidden lines of objects, which do not appear in the image, were eliminated through visibility analysis to reduce search space and to increase the robustness of matching process. In [22], the work of [21] was extended to increase the robustness of the matching by devising a rule-based grouping method. However, a shortcoming of the approach is that matching fails in cases of nadir images where building walls and footprints are invisible in the image. Similar method was used to match 3D building models to aerial images by [23]. However, their implementation was only tested for a small number of buildings and it was limited to standard gable roof models. Also, a requirement of their approach was that each pixel must be within some range of an edge. 2D orthogonal corner (2DOC) was used in [24] as a feature to recover the camera pose for texture mapping of 3D building model. The coarse camera parameters were determined by vertical vanishing points that correspond to vertical lines in the 3D models. Correspondences between image 2DOC and DSM 2DOC were determined using Hough transform, and generalized M-estimator sample consensus. However, they described their error source as too limited to correct 2DOCs matches, in particular, for residential areas. Instead of using 2DOC, Wang *et al.* [25] proposed three connected segments (3CS) as a feature, which is more distinctive, and repeatable. For putative feature matches, they applied a two level RANSAC method, which consists of a local, and a global RANSAC for robust matching.

## 2. Registration Method

**Figure 1:**
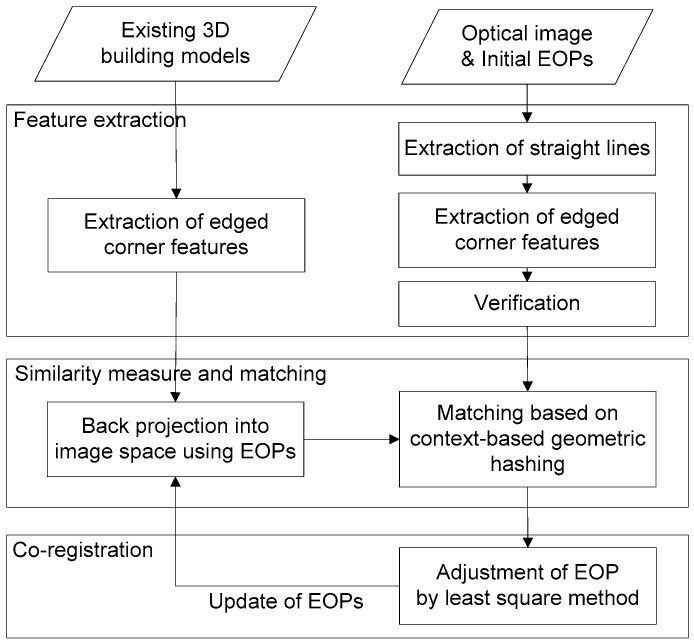
Flowchart of the proposed model-to-image registration method.

Figure 1 illustrates the proposed method for registering a single image with existing 3D building models using extracted edged corner features. It starts by back-projecting the 3D building models to the image using initial (or at later steps updated) EOPs. Then with the help of the similarity measure, the matching process finds corresponding features using a CGH method. Based on the matched feature pairs, the EOPs of the single image are estimated by a least square adjustment. As shown in Figure 1, the second and third steps are conducted iteratively to find optimal EOPs until the corresponding matching pairs do not further improve. The three steps of the proposed method are further discussed in the following sub-sections whereat the last two steps are discussed together.

### 2.1. Feature Extraction

Feature extraction is the first step of the registration task. As previously mentioned, feature selection should consider the properties of the given datasets, the application, and the required accuracy. In this study, we use two different types of features: edged corner features, and context features. An edged corner feature, which consists of a corner point, and the two associated lines that potentially intersect at this point (“arms”), provides local structure information for a building. In building models, it is relatively straightforward to extract this feature because each vertex of a building polygon can be treated as a corner and the connected lines as arms. Note that only rooftop polygons are considered for this. In an image with rich texture information, various corner detectors, and line detectors can be used to extract edged corner features. A context feature is defined as a characteristic spatial relation between two edged corner features selected within an individual roof. This context feature is used to represent global structure information so that more accurate, and robust matching results can be achieved. Section 2.1.1 explains the extraction of edged corner features from an image, and Section 2.1.2 describes the properties of context features.

#### 2.1.1. Edged Corner Feature Extraction from Image

**Figure 2:**
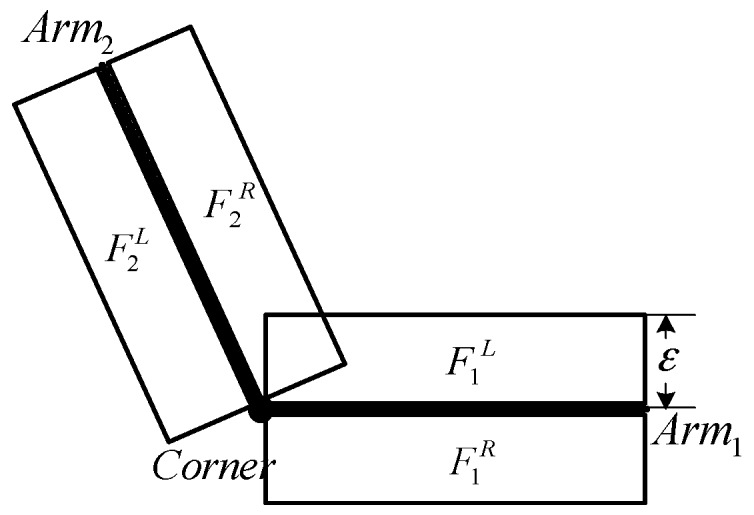
Edged corner feature (corner and its arms) and flanking regions.

Edged corner features from a single image are extracted by three separate steps; (1) extraction of straight lines; (2) extraction of corners, and their arms; and (3) verification. The process starts with the extraction of straight lines from a single image by applying a straight line detector. We use Kovesi’s algorithm, which relies on the calculation of phase congruency to localize, and link edges [26]. Then, corners are extracted by estimating the intersection of the extracted straight lines, considering the proximity with a given distance threshold ($T_{d}=20\text{ pixels}$). Afterwards, corner arms are determined by two straight lines used to extract the corner with fixed length (20 pixels). This procedure may produce incorrect corners because the proximity constraint is the only one considered. Thus, the verification process removes incorrectly extracted corners based on geometric and radiometric constraints. As a geometric constraint, the inner angle between two corner arms is calculated, and investigated to remove corners with sharp inner angles. In general, many of building structures appears in regular shapes following orthogonality and parallelism where small acute angles are found to be uncommon. Through this process, incorrectly extracted corners are filtered out by applying a user-defined inner angle threshold ($T_{\theta }=10{}^{\circ}$). For the radiometric constraint, we analyze the radiometric values (Digital Number (DN) value or color value) of the left, and right flanking regions ($F_{1}^{L},F_{1}^{R},F_{2}^{L},F_{2}^{R}$) of each corner arm with a flanking width (*ε*) as used in [27]. Figure 2 shows a configuration of a corner, its arms, and the concept of the flanking regions. In a correctly extracted corner, the average DN (or color) difference between $F_{1}^{L}$ and $F_{2}^{R}$, $∥F_{1}^{L}-F_{2}^{R}∥$, or between $F_{1}^{R}$ and $F_{2}^{L}$, $∥F_{1}^{R}-F_{2}^{L}∥$, is likely to be small, underlining the homogeneity of two regions, while average DN difference between $F_{1}^{L}$ and $F_{2}^{L}$, $∥F_{1}^{L}-F_{2}^{L}∥$, or between $F_{1}^{R}$ and $F_{2}^{R}$, $∥F_{1}^{R}-F_{2}^{R}∥$, should be large enough to underline the heterogeneity of two regions. Thus, we measure two radiometric properties: the minimum average DN difference value of two neighbor flanking regions for homogeneity measurement, $D_{min}^{homo}=min\left(∥F_{1}^{L}-F_{2}^{R}∥,∥F_{1}^{R}-F_{2}^{L}∥\right)$, and the maximum DN difference value of two opposite flanking regions for heterogeneity measurement, $D_{max}^{hetero}=max\left(∥F_{1}^{L}-F_{2}^{L}∥,∥F_{1}^{R}-F_{2}^{R}∥\right)$. A corner is considered an edged corner feature if the corner has a smaller $D_{min}^{homo}$ than a threshold $T_{homo}$, and if it has a larger $D_{max}^{hetero}$ than a threshold $T_{hetero}$.

In order to determine thresholds for two radiometric properties, we assume that the intersection points are generated from both correct corners, and incorrect corners; and the two types of intersection points have different distributions with regards to their radiometric properties. Because there are two cases (correct corner and incorrect corner) for the average DN difference values, we can use the Otsu’s binarization method [28] to automatically determine an appropriate threshold value. The method was originally designed to extract an object from its background for binary image segmentation based on histogram distribution. It calculates the optimum threshold by separating the two classes (foreground and background) in such a way that their intra-class variance is minimal. In our study, a histogram of homogeneity values (or heterogeneity values) for the entire selection of points is generated, and the optimal threshold for homogeneity (or heterogeneity) is automatically determined by Otsu’s binarization method.

#### 2.1.2. Context Features

**Figure 3:**
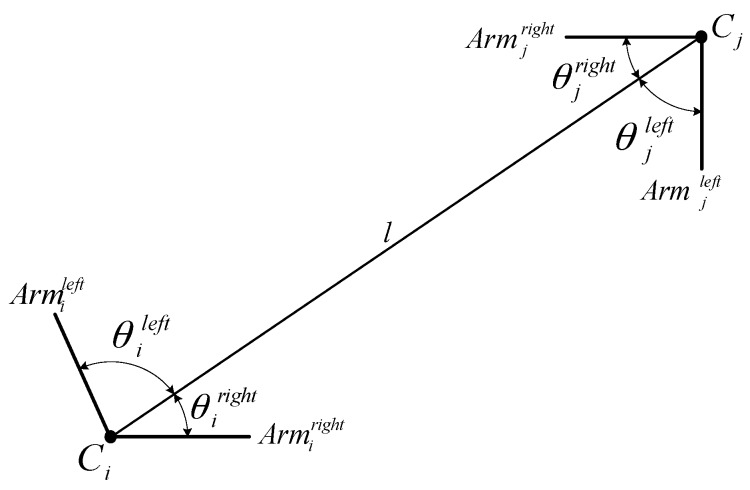
Context feature.

While an edged corner feature provides only local structure information about a building corner, context features partly impart global structure information related to the building configuration. Context features are set by selecting any two adjacent edged corner features, that is, four angles ($\theta_{i}^{left},\theta_{i}^{right},\theta_{j}^{left},\theta_{j}^{right}$) between a line (*l*) connecting the two corners ($C_{i}$ and $C_{j}$), and their arms ($Arm_{i}^{\text{left}},Arm_{i}^{right},Arm_{j}^{left},Arm_{j}^{\text{right}}$) as shown in Figure 3. Note that each angle is determined by the relative line connecting any two corners (*l*). The context feature, which is invariant under scale, translation, and rotation, is used to calculate contextual similarity in our proposed score function (see Section 2.2.2).

### 2.2. Similarity Measurement and Matching

Similarity measurement and matching process take place in the image space after the 3D building models are back-projected onto the image space using the collinearity equations with the initial EOPs (or updated EOPs). In order to find reliable and accurate correspondences between features extracted from a single image, and building models, we introduce a CGH method where the vote counting scheme of a standard geometric hashing is supplemented by a newly developed similarity score function. The similarity score function consists of a unary term, and a contextual term. The unary term measures the similarity between edged corner features derived from the image, and models while the contextual term measures the geometric property of context features. In the following sections, the standard geometric hashing, and its limitations are described (Section 2.2.1), and our proposed CGH method is introduced (Section 2.2.2).

#### 2.2.1. Geometric Hashing

**Figure 4:**
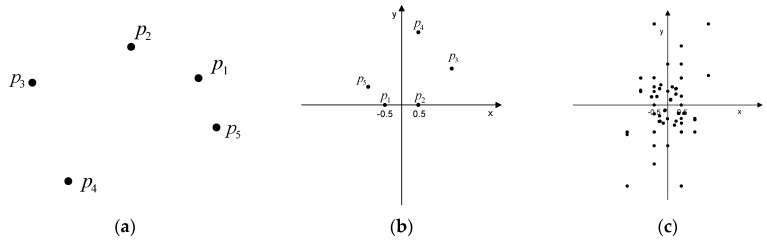
Geometric Hashing: (**a**) model points; (**b**) hashing table with base pair and (**c**) all hashing table entries with all base pairs.

Geometric hashing, a well-known indexing-based approach, is a model-based object recognition technique for retrieving objects in scenes from a constructed database [29]. In geometric hashing, an object is represented as a set of geometric features such as points, and lines, and by its geometric relations, which are transformation-invariant under certain transformations. Since only local invariant geometric features are used, geometric hashing can handle partly occluded objects. Geometric hashing consists of two main stages: the pre-processing stage, and the recognition stage. The pre-processing stage encodes the representation of the objects in a database, and stores them in a hash table. Given a set of object points ($p_{k};k=0,\ldots ,n$), a pair of points ($p_{i}$ and $p_{j}$) is selected as a base pair (Figure 4a). The base pair is scaled, rotated, and translated into the reference frame. In the reference frame, the magnitude of the base pair equals 1; the midpoint between $p_{i}$ and $p_{j}$ is placed at the origin of the reference frame. The vector $\left(\vec{p_{i}p_{j}}\right)$ corresponds to a unit vector of the *x*-axis. The remaining points of the model are located in the coordinate frame based on the corresponding base pair (Figure 4b). The locations (to be used as index) are quantized by a proper bin size and recorded with the form (model ID, used base pair ID) in the hash table. For all possible base pairs, all entries of points are similarly recorded in the hash table (Figure 4c).

In the subsequent recognition stage, the invariants, which are derived from geometric features in a scene, are used as indexing keys to assess the previously constructed hash table so that they can be matched with the stored models. In a similar way to the preprocessing stage, two points from a set of points in the scene are selected as the base pair. The remaining points are mapped to the hash table, and all entries in the corresponding hash table bin receive a vote. Correspondences are determined by a vote counting scheme, producing candidate matches.

Although geometric hashing can solve matching problems of rotated, translated, and partly occluded objects, it has some limitations. The first limitation is that the method is sensitive to the bin size used for quantization of the hash table. While a large bin size in the hash table cannot separate between two close points, a small bin size cannot deal with the position error of the point. Secondly, geometric hashing can produce redundant solutions due to its vote counting scheme [29]. Although it can significantly reduce candidate hypotheses, a verification step or additional fine matching step is required to find optimal matches. Thirdly, geometric hashing has a weakness in cases where the scene contains many features of similar shapes at different scales, and rotations. Without any constraints (e.g., position, scale and rotation) based on prior knowledge about the model, geometric hashing may produce incorrect matches due to the matching ambiguity. Fourthly, the complexity of processing increases by the number of base pairs, and the number of features in the scene [6]. To address these limitations, we enhance the standard geometric hashing by changing the vote counting scheme to a score function, and by adding several constraints such as scale difference of a base, and specific selection of bases.

#### 2.2.2. Context-Based Geometric Hashing (CGH)

**Figure 5:**
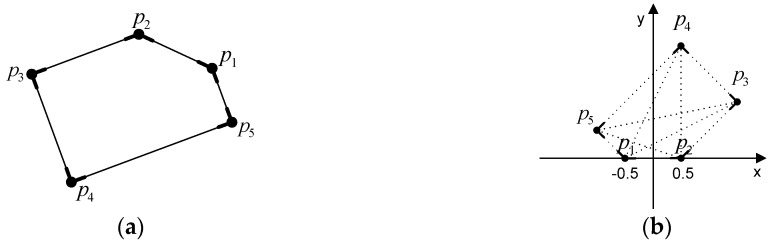
(**a**) Edged corner features derived from a model, and (**b**) information to be stored in a hashing table (dotted lines represent context features).

In this section, we describe the building model objects and the scene by sets of edged corner features. Edged corner features derived from input building models are used to construct the hash table in the pre-processing stage while edged corner features derived from the single image are used in the recognition stage. Each given building model consists of several planes. Thus, in the pre-processing stage, we select two edged corner features, which belong to the same plane of the building model as the base pair. It can reduce the complexity of the hashing table, and ensures that the base pair retains the spatial information of the plane. The selected base pair is scaled, rotated, and translated to define the reference frame. The remaining edged corner features which belong to the whole building model are also transformed with the base pair. In contrast to the standard geometric hashing, our hashing table contains model IDs, feature IDs of the base pair, the scale of the base pair (the rate of real distance of base pair), an index for member edged corner features, and context features generated by combinations with edged corner features. Figure 5 shows an example of the information to be stored in a hashing table.

**Figure 6:**
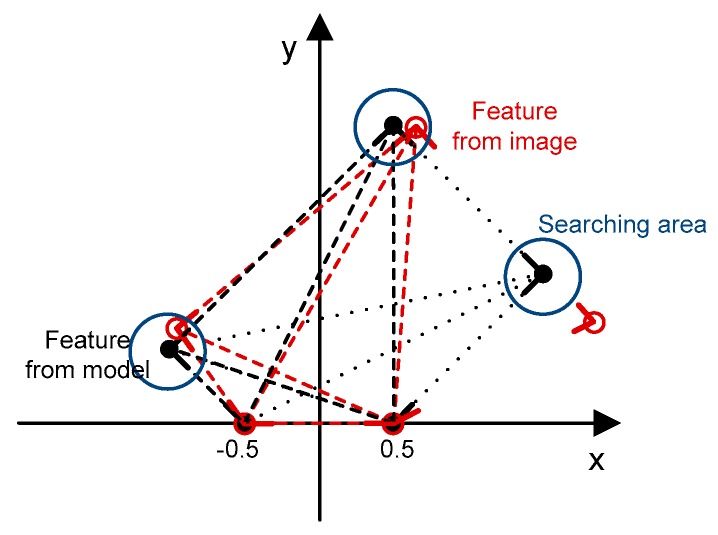
Context features to be used for calculating score function.

Once all possible base pairs are set, the recognition stage tries to retrieve corresponding features based on the designed score function. Two edged corner features from the image are selected as base pair with two constraints: (1) scale constraint; and (2) position constraint. As a constraint on a scale, only those base pairs whose scale is similar to the scale of the base pair in the hash table are considered with an assumption that the initial EOPs provide an approximate scale of the image. Thus, if the scale ratio is smaller than a user defined threshold ($T_{s}=0.98$), the base pair is excluded from the set of possible base pairs. In addition to scale constraint, the possible positions of a base pair can be also restricted with a proper searching space. This searching space can be determined by calculating error propagation with the amount of assumed errors (calculated by the iterative process) for initial EOPs (updated EOPs) of the image, and the models. These two constraints reduce the matching ambiguity, and the complexity of processing. After the selection of possible base pairs from the image, all remaining edged corner features in the image are transformed based on a selected base pair. Afterwards, the optimal matches are determined by comparing a similarity score. The process starts by generating context features from the model, and the image in a reference frame. Given a model that consists of five edged corner features (black color), ten context features can be generated as shown in Figure 6. Note that all edged corner features derived from the model are not matched with edged corner features derived from the image (red color). Thus, only edged corner features, which have corresponding image edged corner features within the search area (*n* = 4 in Figure 6), and their corresponding context features (*m* = 6 in Figure 6 (red long-dash)) are considered in the calculation of the similarity score function.

The newly designed score function consists of a unary term, which measures the position differences of the matched points, and a contextual term, which measures length and angle differences of corresponding context features, as follows:

(1) $$score=\alpha \times \left[w\times \frac{\sum_{i=1}^{n}U\left(i\right)}{n}+\left(1-w\right)\times \frac{\sum_{i=1}^{n}\sum_{j=1}^{n}C\left(i,j\right)}{m}\right]$$

where:

(2) $$\alpha =\{\begin{array}{l} 0\;if\;\frac{\#\;of\;matched\;features}{\#\;of\;features\;in\;the\;model}<T_{c}\\ 1\;else \end{array}$$

α is an indicator function where the minimum number of features to be matched is determined depending on $T_{c}$ ($T_{c}=0.5$, at least 50% of corners in the model should be matched with corners from the image) so that all features of the model do not need to be detected in the image; *n* and *m* are the number of matched edged corner features, and context features, respectively; *w* is a weight value which balances the unary term and the contextual term; in our case, *w* = 0.5 is heuristically selected:

*Unary term*: The unary term U(i) measures the position distance between edged corner features derived from the model, and the image in a reference frame. The position difference $∥P_{i}^{M}-P_{i}^{I}∥$ between an edged corner feature in the model and its corresponding feature in the image is normalized by the distance $N_{i}^{P}$ calculated by the EOP error propagation on the image plane:

(3) $$U\left(i\right)=\frac{N_{i}^{P}-∥P_{i}^{M}-P_{i}^{I}∥}{N_{i}^{P}}$$

*Contextual term*: This term is designed to measure the similarity between context features in terms of length and four angles. The contextual term is calculated for all context features which are generated from matched edged corner features. For the length difference,$\;∥L_{ij}^{M}-L_{ij}^{I}∥$, the difference between lengths of context features in the model, and in the image is normalized by length $N_{ij}^{L}$ of the context feature in the model. For angle differences, the angle difference $∥\theta_{ij}^{M_{k}}-\theta_{ij}^{I_{k}}∥$ between the inner angles of a context feature is normalized by $N_{ij}^{\theta }$ ($N_{ij}^{\theta }=\frac{\pi }{2}$):

(4) $$C\left(i.j\right)=\frac{N_{ij}^{L}-∥L_{ij}^{M}-L_{ij}^{I}∥}{N_{ij}^{L}}+\frac{\sum_{k=1}^{4}\left(N_{ij}^{\theta }-∥\theta_{ij}^{M_{k}}-\theta_{ij}^{I_{k}}∥\right)}{4\times N_{ij}^{\theta }}$$

For each model, a base pair, and its corresponding corners which maximize the score function are selected as optimal matches. Note that if the maximum score is smaller than a certain threshold $T_{m}$, the matches are not considered as matched corners. The role of $T_{m}$ is to determine an optimal subset of accurate matching correspondences for estimating EOP parameters. High $T_{m}$ values provide a low number of matching correspondences with high accuracy. In contrast, low $T_{m}$ values increase the number of matching correspondences but they also decrease their accuracy. Once all correspondences are determined, the EOPs of the image are adjusted through space resection using pairs of object coordinates of the existing building models, and newly derived image coordinates from the matching process. Values calculated from the similarity score function are used to weight matched pairs. The process continues until matched pairs do not change.

## 3. Experimental Results

**Figure 7:**
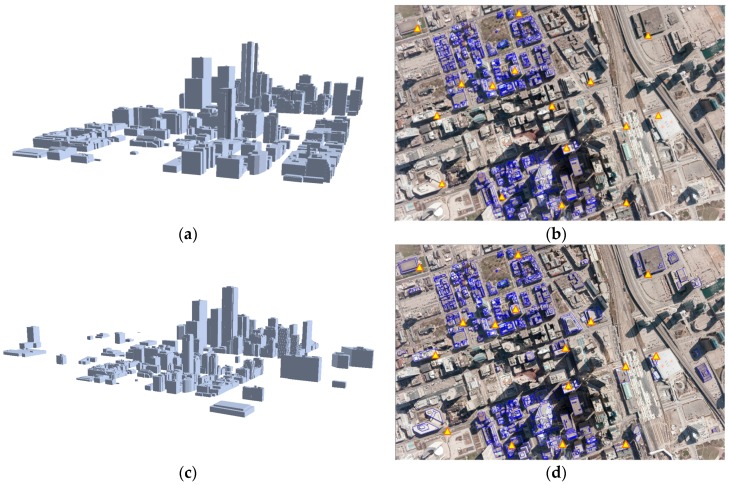
Toronto dataset: (**a**) LiDAR-driven building models reconstructed by [31]; (**b**) LiDAR-driven building models (blue lines) and check points (yellow triangles) back-projected to image; (**c**) manually digitized building models and (**d**) manually digitized building models back-projected to image.

**Figure 8:**
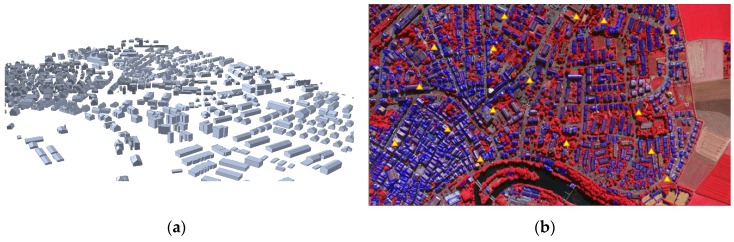
Vaihingen dataset: (**a**) LiDAR-driven building models reconstructed by [33] and (**b**) LiDAR-driven building models (blue lines) and check points (yellow triangles) back-projected to image.

The proposed CGH-based registration method was tested on benchmark datasets over the downtown areas in Toronto (ON, Canada) and Vaihingen in Germany provided by the ISPRS Commission III, WG3/4 [30]. Table 1 shows characteristics of reference building models, which were used to determine EOPs. For the Toronto datasets, two different types of reference building models were prepared by: (1) a manual digitization process conducted by human operators; and (2) using a state-of-the art algorithm [31] from airborne LiDAR point clouds. These two building models were used to investigate their respective effects on the performance of our method (Figure 7). For the Vaihingen datasets, LiDAR-driven building models were automatically generated by [32] and adjusted as described in [33] as shown in Figure 8. A total of 16 check points for each dataset, which were evenly distributed throughout the images, were used to evaluate the accuracy of the EOPs.

**Figure 9:**
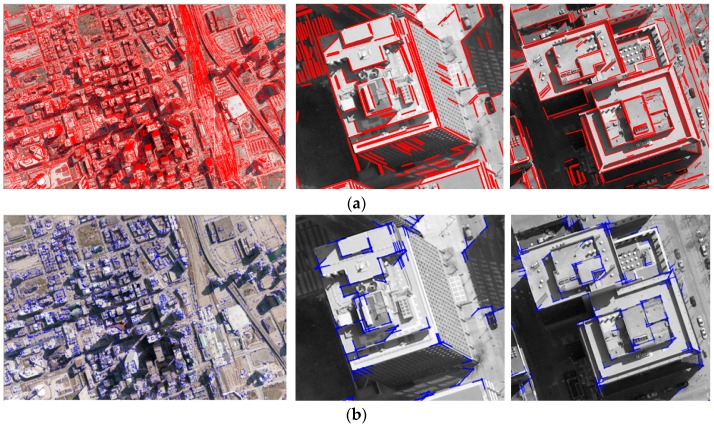
Edged corner features from image: (**a**) straight lines (red) and (**b**) edged corner features (blue).

For the Toronto dataset, various analyses were conducted to evaluate the performance of the proposed registration method in detail. From the aerial image, a total of 90,951 straight lines were extracted and 258,486 intersection points were derived by intersecting any two straight lines found within 20 pixels of proximity constraint. Out of these, 57,767 intersection points were selected as edged corner features following the removal of 15%, and 60% of intersection points using geometric constraint ($T_{\theta }=10{}^{\circ}$), and radiometric constraints ($T_{homo}=26$, and $T_{hetero}=55$), respectively (Table 2). The $T_{homo}$ and $T_{hetero}$ were automatically determined by Otsu’s binarization method. Figure 9 shows edged corner features extracted from the aerial image. As many of the intersection points are not likely to be corners, the majority of them were removed. The method correctly detected corners and arms in most cases even though some corners were visually difficult to detect due to their low contrasts.

**Figure 10:**
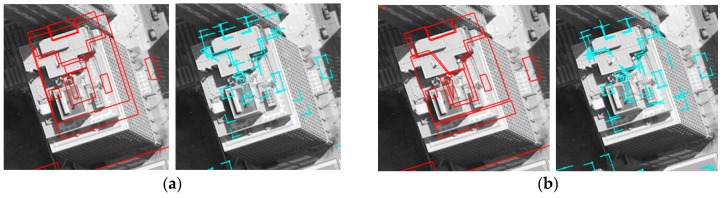
Features from existing building models: (**a**) manually digitized building models and their edged corner features and (**b**) LiDAR-driven building models and their edged corner features.

After the existing building models were back-projected onto the image using error-contained EOPs, edged corner features were extracted from the vertices of the building models in the image space (Figure 10). It should be noted that two different datasets were used as the existing building models. Some edged corner features extracted from both existing building models were not observed in the image due to occlusions caused by neighbor building planes. Also, some edged corner features, in particular those extracted from LiDAR-driven building models, do not match with the edged corner features extracted from the image due to modeling errors caused by irregular point distribution, occlusions and the reconstruction mechanism. Thus, correspondences between edged corner features from the image and from the existing building models are likely to be partly established.

The proposed CGH method was applied to find correspondences between features derived from the image and from existing building models. When manually digitized building models are used as building models, a total of 693 edged corner features (7.8% of edged corner features extracted from the entire building models) were matched using the parameters given in Table 3.

It is noted that only models whose vertices were greater than $T_{c}$ were considered to find possible building matches. For LiDAR-driven building models, only 381 edged corner features (4.9% from the entire building models) were matched (Table 2). It is noted that the number of matched edged corner features is influenced by the quality of the existing building models, and thresholds used, $T_{m}$ in particular. As shown in Table 2, more edged corner features are matched when manually digitized building models were used as the existing building models than when LiDAR-driven building models were used. If $T_{m}$ is set as a small value, the number of matched edged corner features increases, but this increases the risk it may contain a large number of incorrect matched edged corner features. The effect on the $T_{m}$ will be discussed in detail later.

**Figure 11:**
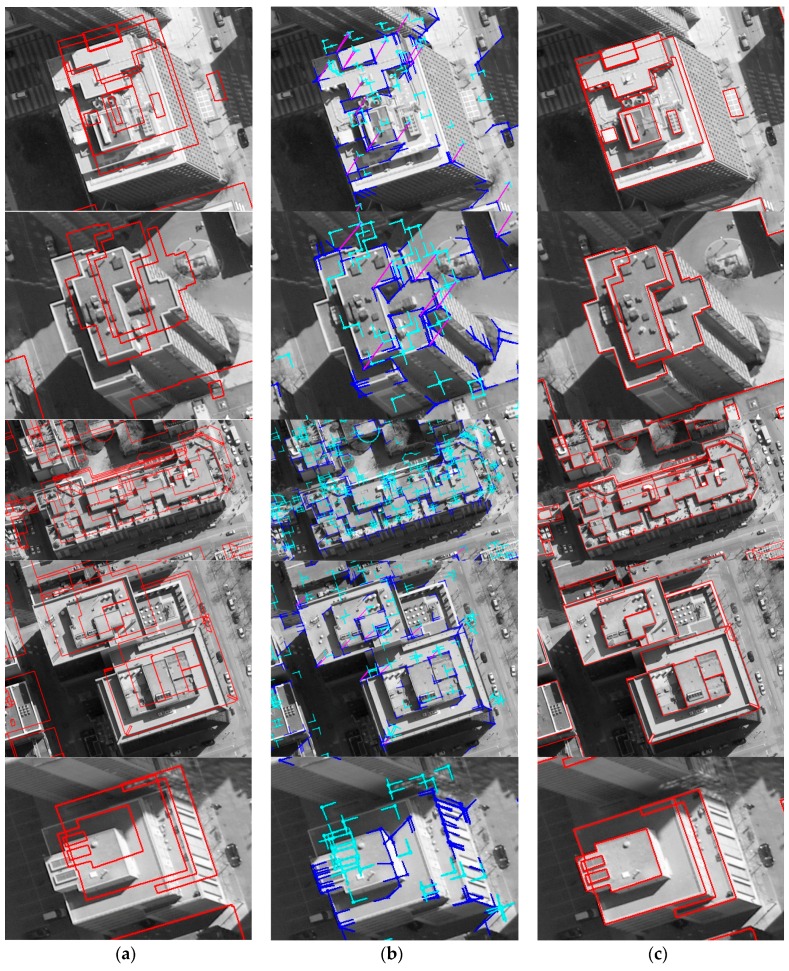
Manually digitized building models: (**a**) with error-contained EOPs; (**b**) matching relations (purple) between edged corner features extracted from the image (blue) and from the models (cyan), and (**c**) with refined EOPs.

**Figure 12:**
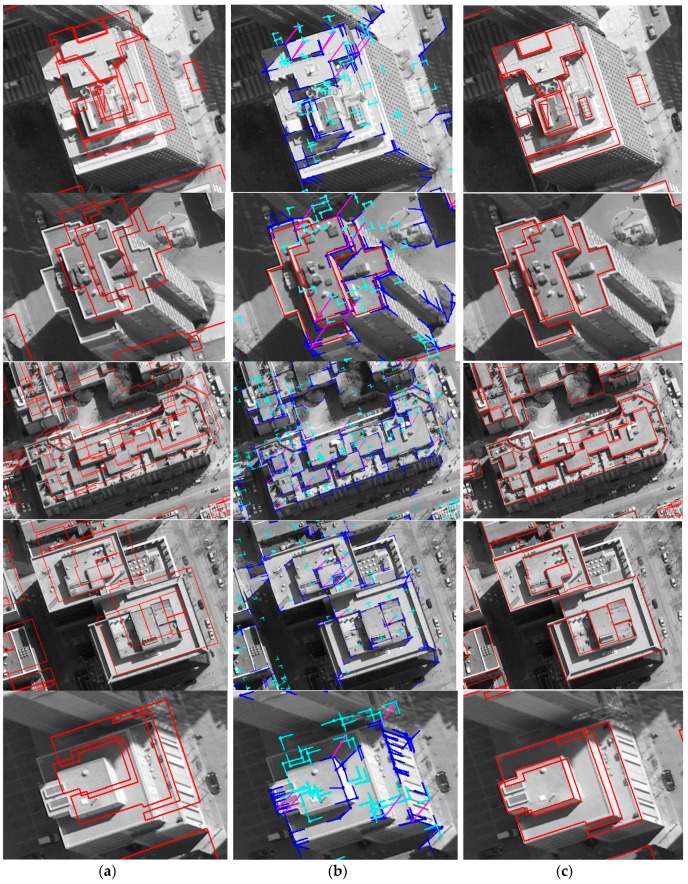
LiDAR-driven building models: (**a**) with error-contained EOPs; (**b**) matching relations (purple) between edged corner features extracted from the image (blue) and from the models (cyan), and (**c**) with refined EOPs.

Based on matched edged corner features, EOPs for the image were calculated by applying the least square method based on co-linearity equations. For qualitative assessment, the existing models were back-projected to the image with refined EOPs. Each column of Figure 11 and Figure 12 shows back-projected building models with error-contained EOPs (a), matched edged corner features (b), and back-projected building models with refined EOPs (c). In the figures, boundaries of the existing building models are well matched to building boundaries in the image with refined EOPs.

In our quantitative evaluation, we assessed the root mean square error (RMSE) of check points back-projected onto the image space using refined EOPs (Table 4). When reference building models were used as the existing building models, the results show that the average difference in *x* and *y* directions are −0.27 and 0.33 pixels, respectively, with RMSE of ±0.68 and ±0.71 pixels, respectively. The results with LiDAR-driven buildings models show that the average differences in *x* and *y* directions are −1.03 and 1.93 pixels, with RMSE of ±0.95 and ±0.89 pixels, respectively. Although LiDAR-driven building models are used, the accuracy of the EOPs is less than 2 pixels in image space (approximately 30 cm in ground sample distance (GSD)). Considering that the point space (resolution) of the input airborne LiDAR dataset is larger than 0.3 m, the refined EOPs provide a greater accuracy for engineering applications.

**Figure 13:**
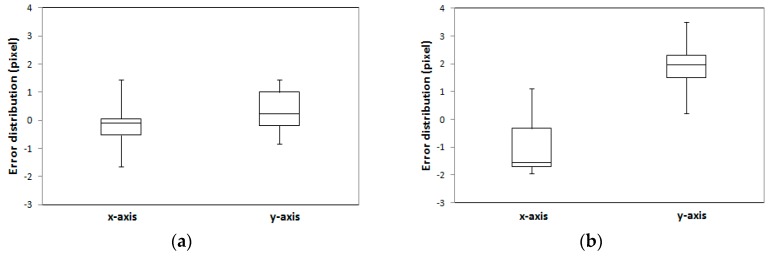
Error distributions for 16 check points when (**a**) manually digitized building models are used and (**b**) LiDAR-driven building models are used.

The error distribution of 16 check points is illustrated in Figure 13. The error distributions showed that the interquartile range (IQR) for both manually digitized and LiDAR-driven building models were under 1.5 pixels. The maximum error value for LiDAR-driven models was however 1 pixel greater than for manually digitized models.

In this study, threshold, $T_{m}$ has an effect on the accuracy of the EOPs. In order to evaluate the effect of $T_{m}$, we estimated the matched number of edged corner features, and calculated the average error and the RMSE of the check points with different values of $T_{m}$. As shown in Table 5, the number of matched features is inversely proportional to the value of $T_{m}$, regardless of which existing building models are used. However, the effect of $T_{m}$ on the accuracy is not the same for both building models. We observed $T_{m}$ affects the matching accuracy of digitized building models less than it does for LiDAR-driven building models. Furthermore, the matching accuracy tends to get worse with very low or high $T_{m}$ values. The latter can be explained by the low number of matched features, giving us insufficient data to accurately adjust the EOPs of the image. In the other case, if a low $T_{m}$ value is selected, the number of matched features increases, but so does the number of incorrect matches if the building models are inaccurate. Thus, we can observe that LiDAR-driven building models, reconstructed with relatively lower accuracy compared to the manually digitized models, produced more sensitive results in the matching accuracy according to $T_{m}$. In contrast, the matching accuracy of the manually digitized building models remains high because of high model accuracy. In summary, a higher accuracy of the building models can lead to a higher EOP accuracy, while the value of $T_{m}$ should be determined by balancing the ratio of correct matched features and incorrect matched features.

In order to evaluate the effect on context feature, we set weight parameter *w* in score function (Equation (1)) as 1 and 0.5, respectively, and then compared the results. When *w* = 1, the score function considers only the unary term without the effect of the contextual term so that the contextual force is ignored. As shown in Table 6, the results show that registration with only unary terms causes considerably low accuracy in both cases. In particular, with LiDAR-driven models, the accuracy is heavily affected. These results indicate that the use of context features has a positive effect on resolving the matching ambiguity and thus improving the EOP accuracy by reinforcing contextual force.

**Figure 14:**
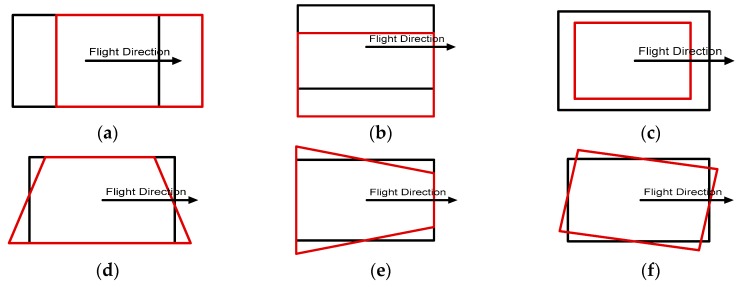
The behaviors caused by errors for EOP parameters: (**a**) $X_{0}$; (**b**) $Y_{0}$; (**c**) $Z_{0}$; (**d**) $\omega_{0}$; (**e**) $\varphi_{0}$; and (**f**) $\kappa_{0}$.

**Figure 15:**
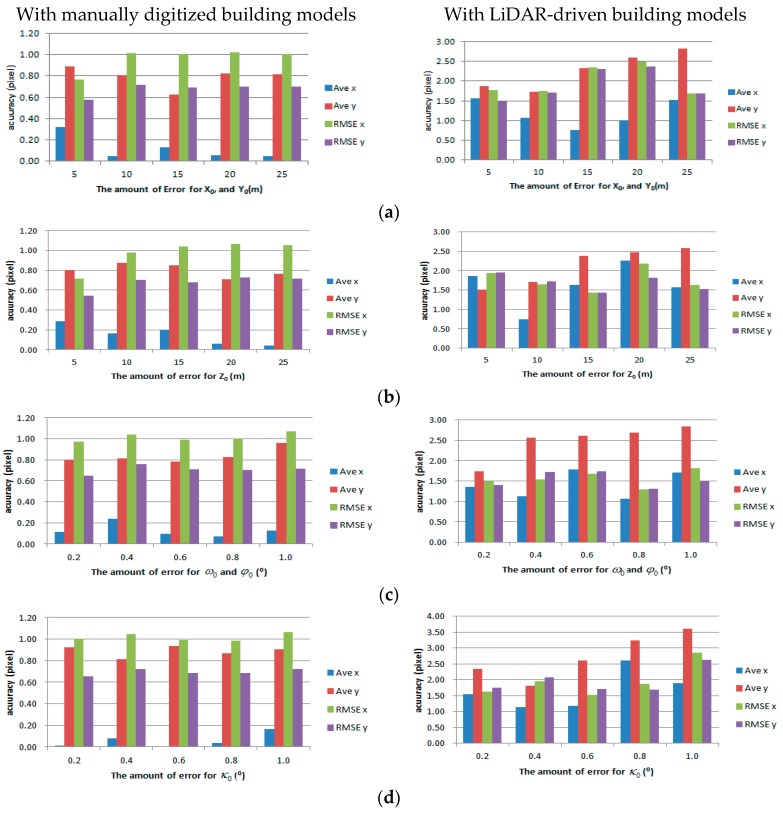
The impacts of errors in initial EOPs: (**a**) $X_{0}$ and $Y_{0}$; (**b**) $Z_{0}$; (**c**) $\omega_{0}$ and $\varphi_{0}$; and (**d**) $\kappa_{0}$.

We also analyzed various impacts of errors in initial EOPs on the matching accuracy by adding different levels of errors to evaluate our proposed method. Each parameter of the EOPs leads to different behaviors from back-projected building models: $X_{0}$ and $Y_{0}$ parameters are related to the translation of back-projected building models; $Z_{0}$ is related to scale; $\omega_{0}$ and $\varphi_{0}$ cause shape distortion; $\kappa_{0}$ is related to rotation (Figure 14). In order to assess the effects on translation and scale, errors ranging from 0 m to 25 m were added to three position parameters. To assess the shape distortion and rotation effects, errors ranging from 0° to 2.5° were added to three rotation parameters. Figure 15 shows the accuracies of the refined EOPs with different level of errors for each EOP parameter. Regardless of errors in the initial EOPs, RMSE of under 2 pixels for manually digitized building models, and RMSE of under 3 pixels for LiDAR-driven building models were achieved. The results indicate that the accuracy of the refined EOPs was less affected by the amount of initial EOPs errors. This is due to the fact that the EOPs converge to the optimum solution iteratively.

**Figure 16:**
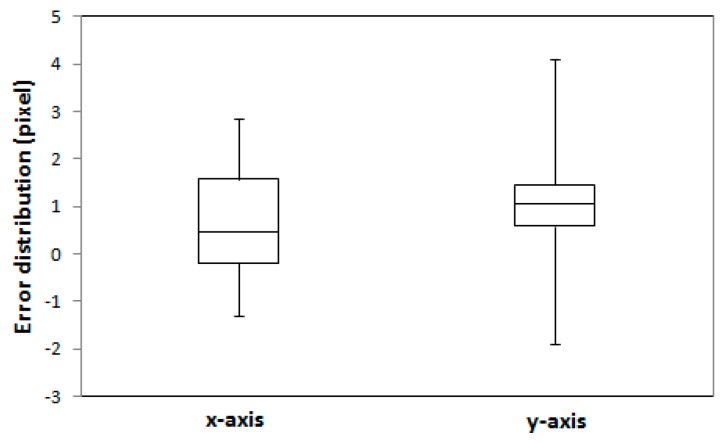
Error distribution of the 16 check points for the Vaihingen dataset.

In order to evaluate the robustness of the proposed registration method, the algorithm was applied to the Vaihingen dataset. A total of 31,072 edged corner features from the image and 11,812 edged corner features from the existing building models were extracted using the parameters set in Table 3. A total of 379 edged corner features were matched by the CGH method where $T_{m}$ was heuristically set as 0.7, and other parameters were set by Table 3. The results of the extracted and matched features are summarized in Table 7. Sixteen check points were evaluated for error-contained EOPs and refined EOPs. The accuracies of the check points with refined EOPs show that the average difference for *x* and *y* directions are 0.67 and 0.91 pixels with RMSE of ±1.25 and ±1.49 pixels respectively (Table 8). A summary of the error distribution for the 16 check points is presented in Figure 16. The results suggest that the proposed registration method can achieve accurate and robust matching results even though building models with different error types were used for the registration of a single image.

## 4. Discussion

In this paper, we proposed a new model-to-image registration method which can align a single image with 3D building models. Edged corner features, represented by a corner and its associated edges, and context features are proposed as the matching features. Edged corner features are extracted from the image by calculating the intersection of two neighboring straight lines, and verified using geometric and radiometric properties. For similarity measurement, and matching, the CGH method was proposed to compensate for the limitations of the standard geometric hashing method. The qualitative assessment showed that the boundaries of the existing building models, back-projected by refined EOPs, are well aligned with boundary lines from the image. Meanwhile, the quantitative assessment showed that both manually digitized building models, and LiDAR-driven building models can be used to evaluate the EOPs of a single image with acceptable and reliable accuracy. More specifically, experimental results are summarized as follows:

- The quality of building models directly affects the accuracy of EOPs. When manually digitized building models were used, the proposed registration method accurately and reliably achieved the EOPs regardless of threshold and assumed error. However, if building models contain more modeling errors, the accuracy of EOPs is reduced, which are more susceptible to threshold, and assumed errors.
- Contextual features employed in geometric hashing enhances matching performance. This is because contextual values provide information about the relation between edged corner features, characterizing geometric properties of individual roof polygon. In particular, the use of context features, which provide global information of building models, that is at larger scale (object-level) than at using single corners only (point-level), plays a significant role in our enhanced geometric hashing method, and making our matching performance more robust to errors involved in building models used.
- The proposed method can iteratively recover the EOPs of a single image in spite of considerable error in their initial values, which exceed error amounts permitted in commercial aerial image acquisition.

As future work, we will extend the proposed method to arbitrarily acquired images (e.g., UAV images, and security camera images).

## Figures and Tables

**Table 1:** Characteristics of reference building models.

Dataset	Reconstruction Method	# of Buildings	# of Planes	Description
Toronto	Manually digitized	159	1560	Complex clusters of high-rise buildings
	LiDAR-driven [31]	126	1066	Maximum building height: approximately 290 m
Vaihingen	LiDAR-driven [33]	894	2619	Typical European style structures with simple building shapes
				Maximum building height: approximately 32 m.

**Table 2:** Extracted features and matched features for the Toronto dataset.

	Image		Existing Building Models	
	Intersections	Corners	Manually Digitized Building Models	LiDAR-Driven Building Models
# of extracted features	258,486	57,767	8895	7757
# of matched features	-	-	693	381

**Table 3:** Parameter setting.

Feature Extraction				Geometric Hashing			
$T_{d}$	$T_{\theta }$	$T_{homo}$	$T_{hetero}$	$T_{s}$	$T_{p}$	$T_{c}$	$T_{m}$
20 pixel	10°	automatic	automatic	0.98	automatic	50%	0.6

**Table 4:** Quantitative assessment with check points (unit: pixel).

Error-Contained Initial EOPs				Refined EOPs with Manually Digitized Building Models				Refined EOPs with LiDAR-Driven Building Models			
Ave.		RMSE		Ave.		RMSE		Ave.		RMSE	
x	y	x	y	x	y	x	y	x	y	x	y
20.51	−24.81	±6.64	±8.22	−0.27	0.33	±0.68	±0.71	−1.03	1.93	±0.95	±0.89

**Table 5:** Effect for $T_{m}$ (unit: pixel).

*T_m_*	Manually Digitized Building Models					LiDAR-Driven Building Models				
	# of Matched Features	Ave.		RMSE		# of Matched Features	Ave.		RMSE	
		x	y	x	y		x	y	x	y
0.9	67	0.38	0.78	±0.43	±0.42	9	0.49	−1.93	±7.39	±6.99
0.8	268	0.00	0.84	±0.81	±0.97	98	−1.09	1.22	±1.53	±1.52
0.7	505	−0.20	0.31	±0.95	±1.08	273	−1.58	1.56	±0.68	±0.61
0.6	693	−0.27	0.33	±0.68	±0.71	381	−1.03	1.93	±0.95	±0.89
0.5	766	−0.22	0.21	±0.81	±0.66	438	−0.43	3.26	±2.61	±3.52
0.4	796	0.25	−0.08	±1.06	±0.75	499	1.21	2.15	±3.06	±3.66
0.3	800	0.00	−0.09	±0.88	±0.71	502	1.37	2.19	±3.12	±3.93
0.2	800	0.00	−0.09	±0.88	±0.71	502	1.37	2.19	±3.12	±3.93
0.1	800	0.00	−0.09	±0.88	±0.71	502	1.37	2.19	±3.12	±3.93

**Table 6:** Effect of context features (unit: pixel).

	Manually Digitized Building Models					LiDAR-Driven Building Models				
	# of Matched Features	Ave.		RMSE		# of Matched Features	Ave.		RMSE	
		x	y	x	y		x	y	x	y
Unary term only (*w* = 1)	542	−0.67	−0.39	±1.56	±1.84	361	5.98	1.17	±7.72	±5.31
Unary term and contextual term (*w* = 0.5)	693	−0.27	0.33	±0.68	±0.71	381	−1.03	1.93	±0.95	±0.89

**Table 7:** Extracted features and matched features (the Vaihingen dataset).

	Image			Model
	Straight Lines	Intersections	Edged Corners	Edged Corners
# of extracted features	276,109	181,200	31,072	11,812
# of matched features	-	-	379	379

**Table 8:** Quantitative assessment with check points (the Vaihingen dataset, unit: pixel).

With Error-Contained Initial EOPs				With Refined EOPs							
				Unary Term Only (*w* = 1)				Unary Term and Contextual Term (*w* = 0.5)			
Ave.		RMSE		Ave.		RMSE		Ave.		RMSE	
x	y	x	y	x	y	x	y	x	y	x	y
22.92	−19.06	±2.28	±3.90	−1.32	−0.35	±2.45	±2.93	0.67	0.91	±1.25	±1.49
